# Serum microRNA 143 and microRNA 215 as potential biomarkers for the diagnosis of chronic hepatitis and hepatocellular carcinoma

**DOI:** 10.1186/1746-1596-9-135

**Published:** 2014-07-02

**Authors:** Zhu-qing Zhang, Hua Meng, Nan Wang, Li-na Liang, Li-na Liu, Shu-ming Lu, Yong Luan

**Affiliations:** 1 Department of Pathology, Dalian Municipal Central Hospital, Dalian 116033, China; 2 Department of Gastroenterology, the First Affiliated Hospital of Dalian Medical University, Dalian 116011, China; 3 Department of Clinical Lab, the First Affiliated Hospital of Dalian Medical University, Dalian 116011, China; 4 Department of Anesthesiology, the First Affiliated Hospital of Dalian Medical University, Dalian 116011, China

## Abstract

**Background:**

Hepatocellular carcinoma (HCC) is regarded as one of the most common malignancies and among the leading causes of cancer death among the whole world. The most urgent needs are to find sensitive markers for early diagnosis or monitor postoperative recurrence and to give adequate treatment for HCC. MicroRNAs (miRNAs) are reported as a group of small non-coding RNAs that can function as endogenous RNA interference to regulate expression of the targeted genes. This study was conducted to detect the application of miR-143 and miR-215 in the diagnosis of HCC.

**Methods:**

A total of 340 serum samples (127 samples from controls, 118 samples from hepatitis and 95 samples from HCC patients) were collected. The levels of the two mature miRNAs (miR-143 and miR-215) were detected by probe-based stem-loop quantitative reverse-transcriptase PCR (RT-qPCR) in controls, hepatitis and HCC patients. Besides, the relationship between miR-143 and miR-215 levels and clinical and pathological factors was explored.

**Results:**

We found that the expression of serum miR-215 was distinctly increased in chronic hepatitis compared with controls (mean ± SD: 6.79 ± 0.72 vs. 3.46 ± 0.37, P < 0.001 and mean ± SD: 8.38 ± 0.87 vs. 3.46 ± 0.37, P < 0.001). In addition, we conduct ROC analyses to detect the potential application of miR-143 and miR-215 in the diagnosis of chronic hepatitis and HCC. Our results showed that miR-143 and miR-215 might be a potential biomarker for the hepatitis and HCC.

**Conclusions:**

In conclusion, the expression of miR-143 and miR-215 in serum were significantly up-regulated in patients with chronic hepatitis and HCC. Due to its reasonable sensitivity and specificity for both diseases, miR-143 and miR-215 could be as potential circulating biomarkers.

**Virtual Slides:**

The virtual slide(s) for this article can be found here: http://www.diagnosticpathology.diagnomx.eu/vs/1048932281272754

## Background

Hepatocellular carcinoma (HCC) is regarded as one of the most common malignancies and among the leading causes of cancer-related deaths among the whole world, especially in East Asia and South Africa [1,2]. HCC ranks the second in China among all malignancies and its mortality is almost equal to its morbidity [3,4]. The carcinogenesis of HCC is a multifactor, multi-step, complex process and it is associated with a background of chronic liver diseases or persistent infection of hepatitis B virus (HBV) or hepatitis C virus (HCV), along with alcohol drinking which are widely recognized etiological agents in HCC. However, the underlying mechanisms that lead to malignant transformation of infected cells remained unclear by now. Most HCC patients died quickly because of the rapid tumor progression and hepatic resection or transplantation is the only potential curative treatment for HCC patients. By now, surgery remains the best prognostic tool for long-term survival of HCC patients; however, more than 80% of patients with HCC have underlying cirrhosis, and of these patients, only 10% to 15% are potentially resectable. The rest are unresectable because of size, location or severity of underlying liver disease. The most urgent needs are to find sensitive markers for early diagnosis or monitor postoperative recurrence and to give adequate treatment for HCC.

Up to now, although serum alpha-fetoprotein (AFP) level is a useful tumor marker for the detection and monitoring of HCC, the false negative rate with AFP level alone may be as high as 40% for patients with early stage HCC. Even in the patients with advanced HCC, the AFP levels may remain normal in 15 ~ 30% of all the patients [5]. New specific markers, such as homeobox gene Barx2, SMG-1, GOLM1 and et al. [6-8] have been developed to improve the sensitivity, specificity, early detection and prediction of prognosis of HCC. However, the overall results have been unsatisfactory [9].

MicroRNAs (miRNAs) are reported as a group of small non-coding RNAs that can function as endogenous RNA interference to regulate expression of the targeted genes [10-12]. To date, more than 1000 human miRNAs have been identified and reported in the RNA database. Considering taht altered expression of some miRNAs contributes to human carcinogenesis, a part of these miRNAs have been reported to be useful as potential biomarkers for diagnosis, prognosis, and personalized therapy of human cancers [13-15]. For example, aberrant miRNA expression has been reported in HCC patients or cell lines [16,17]. Given that blood samples can be easily obtained and have the advantages of minimally invasive continuous in vitrotesting and high reproducibility, determining disease-specific circulating miRNAs to predict and diagnose HCC has become the focus of many studies. A number of researchers have reported the potential clinical application of circulating miRNAs (such as miR-122, miR-125b-5p, miR-223-3p, miR-15b and miR-130b) in the diagnosis and prognosis of HCC [18-21]. Thus, further investigation of aberrant miRNA expression could lead to the discovery of novel miRNA biomarkers for HCC.

The objective of this study was to determine whether altered miR-143 and miR-215 expression detected in the serum could be a useful biomarker for the diagnosis of HCC. Real-time quantitative reverse transcription polymerase chain reaction (RT-qPCR) was performed with the following two goals of exploring whether: [1] serum miR-143 and miR-215 are abnormally expressed in hepatitis and HCC; [2] miR-143 and miR-215 expression in serum might diagnose hepatitis and HCC.

## Methods

### Ethics statement

Written informed consents were obtained from all the participants in this study. The samples were processed under approval of the Human Research Ethics Committee of the Dalian Medical University, Shandong, China. For identification, the samples were codified A (for controls), B (Hepatitis) and C (for HCC) followed by a codified number to protect the privacy of individuals during all the further molecular study.

### Patients and samples

Between January 2013 and October 2013, a total of 340 serum samples (127 samples from controls, 118 samples from hepatitis and 95 samples from HCC patients) were collected at the First Affiliated Hospital of Dalian Medical University and Dalian Municipal Central Hospital. All of the HCC patients were diagnosed by liver biopsy or by the findings of at least two radiological tests of HCC, including abdominal ultrasound, magnetic resonance imaging (MRI), hepatic angiography and contrast-enhanced dynamic computed tomography or by increased AFP (AFP ≥200 μg/mL). Patients with secondary or recurrent tumors, a history of other malignant tumors or being included in other studies were excluded from this study. For the 118 chronic hepatitis cases, the diagnosis was based on the serum test. Serum hepatitis B surface antigens (HBsAg) and anti-HCV antibody were assayed by microparticle enzyme immunoassay using commercial kits to determine hepatitis B or hepatitis C infection. A total of 127 cancer-free controls were attached at the physical examination center in the First Affiliated Hospital of Dalian Medical University and matched with cases by age within 5 years and sex. Controls that had clinical liver diseases were excluded.

All subjects were asked to fill a questionnaire to investigate the demographic characteristics, disease history and the history of cancer and alcohol or tobacco use. The clinical characteristics including tumor differentiation, tumor size, metastasis, Child-Pugh class, chemotherapy and surgery were collected from medical records.

### RNA extraction

Total RNA extraction was performed using a miRcute miRNA isolation kit (Tiangen, China), following the manufacturer’s protocol for serum/plasma samples. Samples were enriched for miRNAs in the extraction process. The yields of total RNA were about 250 ng per 400 ml of serum. The extracted RNA was reverse transcription to cDNA as soon as possible. Reverse transcription was carried out using an all-in-one miRNA first-strand cDNA synthesis kit (Genecopoeia). The operation was conducted following the manufacturer’s protocol for serum/plasma samples.

### Real-time PCR quantification of miRNA expression in serum

The expressions of miRNAs were quantified by TaqMan miRNA assays (Applied Biosystems) following reverse transcription (Tiangen, Chin) of 40 ng RNA. Reactions were loaded onto a 96-well plate and run in duplicate on an ABI 7900 Fast Real-Time PCR System (Applied Biosystems). The reactions were firstly incubated at 50°C for 20 seconds and then 95°C for 10 minutes, followed by 40 cycles of denaturation at 95°C for 15 seconds, then 1 minute of annealing/extension at 60°C. The ΔΔCT method was used to determine relative number of copies (RQ) of miRNA. The U6 was chosen as the endogenous normalizer. The primers used for qRT-PCR in this study are as following: miR-143: GCTGAGATGAAGCACTGAAGCTC, miR-215: AATATTGGCTAGCAGCACGTA and U6: CAAAGTCAGTGCAGGTAGGCTTA.

### Statistical analysis

The statistical analyses in this study was performed using Statistical Program for Social Sciences (SPSS) software 17.0 (SPSS Inc., Chicago, USA). The t-test between two groups were used to analyze the differences between two groups. Nonparametric tests (the Wilcoxon-Mann–Whitney test between two groups and the Kruskall-Wallis test for three or more groups) were used to analyze the relationship between the above miRNA expression level and various clinicopathologic characteristics. The receiver operating characteristic curve (ROC) analysis was undertaken using the expression level for each miRNA in the serum from cases and controls to assess the diagnostic accuracy of each parameter. Using this approach, the area under the ROC (AUC) identified optimal sensitivity and specificity levels at which to distinguish normal individuals. All the *P* values were shown two sided and a *P* value of < 0.05 was considered statistically significant.

## Results

### Baseline and clinicopathologic characteristics of the patients

The baseline and clinicopathologic characteristics of the all the participants are presented in Table 1. The median ages of the control group, chronic hepatitis and HCC patients at diagnosis were 52.58 ± 6.98 years, 53.12 ± 7.24 years, and 54.21 ± 6.95 years. Among the three groups, there are no differences in the rates of hypertension, diabetes, tobacco smoking and alcohol drinking among the three groups (P > 0.05). Among the 95 HCC cases, 49 cases have a size over 5 cm while the others less than 5 cm. The tumor stages are 37 cases of TNM-I, 29 cases of TNM-II, 26 cases of TNM-III and 13 cases of TNM-IV. The lymph nodes and distant metastasis were observed in 12 and 8 cases, respectively.

**Table 1:** Characteristics of hepatocellular carcinoma patients, and controls

**Clinicopathological features**	**No. of participants**	**Groups**			***P***
		**Control (n = 127)**	**Hepatitis (n = 118)**	**HCC (n = 95)**	
**Mean age (year,mean ± SD)**	340	52.58 ± 6.98	53.12 ± 7.24	54.21 ± 6.95	0.634
**Gender**					
Male	184	71	65	60	0.217
Female	156	56	53	35	
**Hypertension**					
No	120	42	42	36	0.129
Yes	220	85	75	59	
**Diabetes mellitus**					
No	302	112	105	85	0.863
Yes	38	15	13	10	
**Tobacco smoking**					
No	192	74	68	62	0.207
Yes	148	53	65	33	
**Alcohol consumption**					
No	238	89	82	77	0.326
Yes	102	38	36	22	
**Tumor size (cm)**					
≥5 cm				49	
<5 cm				46	
**TNM staging**					
TNM-I				37	
TNM-II				29	
TNM-III				16	
TNM-IV				13	
**LN metastasis**					
No				83	
Yes				12	
**Distant metastasis**					
No				87	
Yes				8	

*HCC*: Hepatocellular carcinoma; *LN*: Lymph nodes.

### MiR-143 and miR-215 expression in chronic hepatitis and HCC

**Figure 1:**
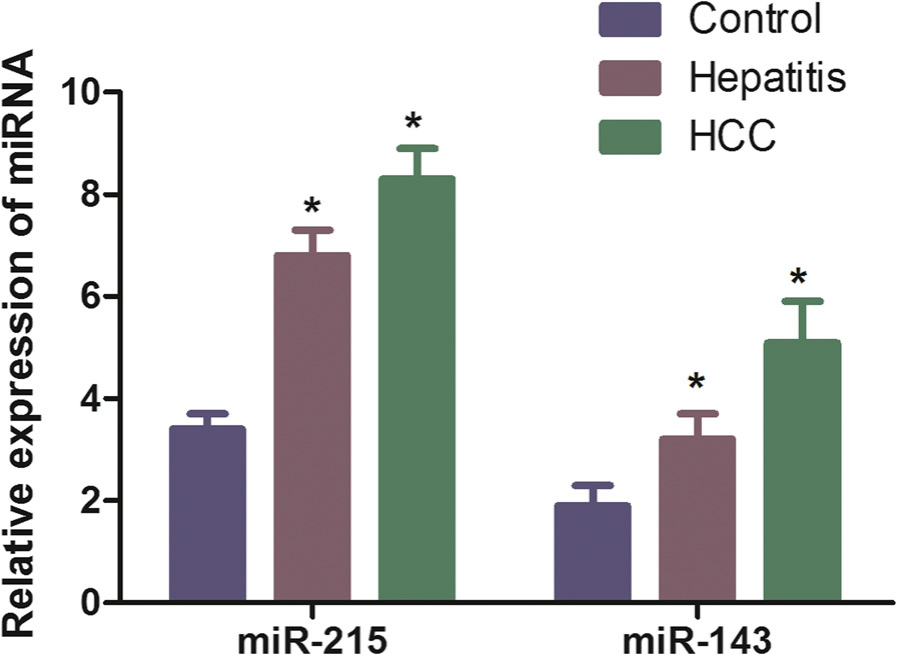
**Relative expression levels of miR-143 and miR-215 in 127 controls, 118 hepatitis and 95 HCC patients.** The expression levels were determined using a qRT-PCR assay, and the relative expression data were analyzed using the 2^- CT^ method. All of the assays were performed in triplicate. U6 was used as an internal control.

Serum miR-143 and miR-215 expression were detected in 118 chronic hepatitis cases, 95 HCC cases and 127 controls normalized to RNU6B. As shown in Figure 1, we found that the expression of serum miR-215 was distinctly increased in chronic hepatitis compared with controls (mean ± SD: 6.79 ± 0.72 vs. 3.46 ± 0.37, P < 0.001). The serum miR-143 was also increased in patients with HCC compared with the controls (mean ± SD: 8.38 ± 0.87 vs. 3.46 ± 0.37, P < 0.001). The expression of serum miR-143 in chronic hepatitis (3.19 ± 0.51) and HCC group (5.13 ± 0.71) are both higher compared with the control group (1.91 ± 0.44) (P < 0.001).

### Diagnostic accuracy of serum miR-143 and miR-215 for chronic hepatitis and HCC

**Figure 2:**
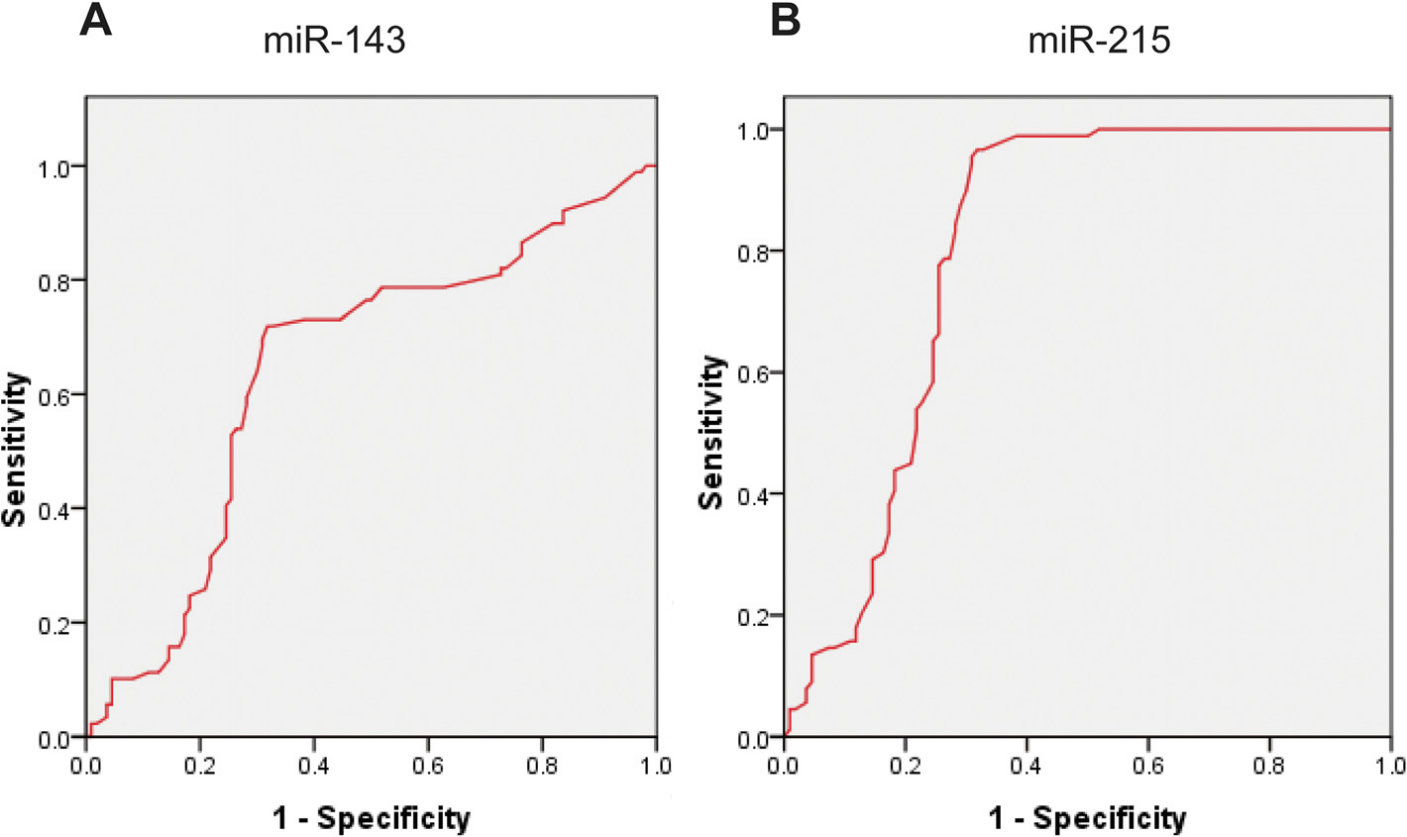
Receiver-operatorcharacteristic (ROC) curves of the A) miR-143 and B) miR-215 for the chronic hepatitis.

**Figure 3:**
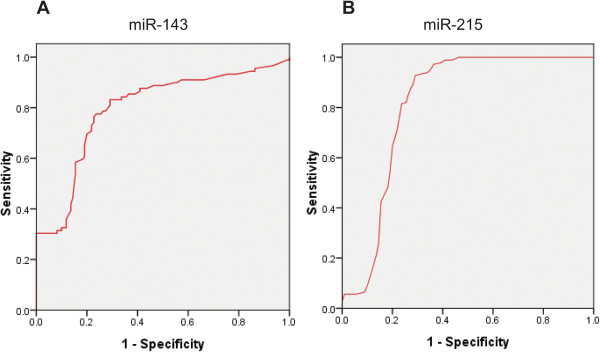
Receiver-operatorcharacteristic (ROC) curves of the A) miR-143 and B) miR-215 for the hepatocellular carcinoma.

The ROC curve analysis was used to analyze the diagnostic accuracy of serum miR-143 and miR-215. ROC curve analyses revealed that both serum miR-143 and miR-215 could serve as valuable biomarkers for chronic hepatitis from healthy controls with an AUC (the areas under the ROC curve) of 0.617 (95% CI: 0.512 – 0.758; P = 0.007) and 0.802 (95% CI: 0.6701 – 0.947; P = 0.0006), respectively (Figures 2A and 2B). At the cut-off value less than 2.14 for miR-143, the sensitivity and the specificity were 78% and 64%, respectively. At the cut-off value less than 4.23 for miR-215, the sensitivity and the specificity were 78% and 89%, respectively.When the diagnostic value of miR-143 and miR-215 for HCC were considered, both of the miRNAs are potential biomarkers for the diagnosis of HCC. The results showed that miR-143 and miR-215 could serve as valuable biomarkers for HCC from healthy controls with an AUC (the areas under the ROC curve) of 0.795 (95% CI: 0.682 – 0.915; P = 0.0001) and 0.816 (95% CI: 0.721 – 0.973; P < 0.0001), respectively (Figures 3A and 2B). At the cut-off value less than 2.21 for miR-143, the sensitivity and the specificity were 73% and 83%, respectively. At the cut-off value less than 4.62 for miR-215, the sensitivity and the specificity were 80% and 91%, respectively.

## Discussion

In this present study, we analyzed the expression of two miRNAs, miR-143 and miR-215, in the serum among the patients with healthy controls, chronic hepatitis and HCC patients. We found that miR-143 and miR-215 expression was up-regulated in the serum samples from patients with chronic hepatitis and HCC compared with the controls. In addition, we conduct ROC analyses to detect the potential application of miR-143 and miR-215 in the diagnoses of chronic hepatitis and HCC. Our results showed that miR-143 and miR-215 might be potential biomarkers for both hepatitis and HCC. Our current data indicated that serum miR-143 and miR-215 expression might be further evaluated as novel noninvasive diagnostic biomarkers for HCC.

A lot of previous studies reported that miRNA expression is aberrant through HCC development; however, most of these studies focused on the expression of miRNAs in HCC tissues and cell lines. For example, a previous study focused on microRNA expression profiles in human hepatitis B virus-related HCC. Based on the data of 11 pairs of HCC and matched non-tumorous tissues from 11 HBV infection patients, miR-96, miR-183, and miR-196a were up-regulated significantly, while let-7c and miR-138 were down-regulated [22]. Xiao et al. conduct a follow-up study and the results revealed that miR-200a was frequently downregulated in HCC. In addition, multivariate analysis confirmed that miR-200a was significantly associated with the overall survival of HCC patients [23]. In an other study, Yu et al. found that miR-424 was down-regulated in HCC cell lines compared a normal hepatocytes and in vitro studies showed that miR-424 is effective in the neoplasty of HCC [24].

Although tissue miRNAs can provide an accurate diagnosis for various types of cancer, the difficulty in collecting tissue samples limits its application for the detection of cancer biomarkers. Acquiring tissue samples is an invasive procedure and depends on surgical sections after initial clinical classification [25]. The search for noninvasive tools for the diagnosis of cancer has long been a goal of many researchers, and much of the interest has been on the circulation of nucleic acids in plasma and serum. Compared with DNA and mRNA, circulating miRNAs show remarkable stability after prolonged incubation at room temperature and/or multiple freezing-thawing processes. However, the protective mechanism of circulating miRNAs is still unknown. Some investigators reported that circulating miRNAs were in the form of argonaute 2 (Ago2)-miRNA complexes that could avoid RNase digestion [26].

Nowadays, there are increasing studies being conducted to identify specific circulating miRNAs in the diagnosis of HCC. Giray et al. conducted a study to investigate the potential of certain serum/plasma miRNAs as novel non-invasive biomarkers for early diagnosis of HBV related HCC. Through comprehensive tests of 94 plasma samples (28 control and 66 patient plasma samples), a series of related miRNAs were reported in the study and the expression of miR-223 was the most significant. Microvesicles (MVs) packaged with miRNAs were reported to be released mainly from tumor cells. Sun et al. conduct expression profiles to detect the differently expressed miRNAs. The results showed that a total of 242 aberrantly expressed miRNAs were identified in HCC-MVs compared with CHB-MVs and the control. Among them, 115 miRNAs were up-expressed with up to 31 fold difference (miR-671-5p) and 127 were down-expressed with up to 0.041 fold difference (miR-432) in HCC [27]. A potential correlation was also evaluated between miR-101 expression and the clinicopathological features and prognosis of HCC patients. It was reported that miR-101 was down-regulated in HBV-related HCC tissues compared with adjacent noncancerous tissues. Furthermore, the miR-101 levels in these tissues from HCC patients were significantly lower than those in tissues from control subjects [28].

In this present study, we focus on the diagnostic value of miR-143 and miR-215 for the chronic hepatitis and HCC. Previous reports have shown that the expression of miR-143 is extremely down-regulated in colorectal cancer, lung, bladder, and gastric cancers [29-31]. While it is reported the expression of miR-143 is up-regulated in pancreatic stellate cells cancer and esophageal cancer [32,33]. There are few reports about the relationship between miR-143 expression and the diagnosis of HCC. Up to now, only one study reported the association between miR-143 and HCC. Zhang et al. reported that the levels of miRNA-143 (miR-143) are dramatically increased in metastatic HBV-HCC of both p21-HBx transgenic mice and HCC patients. Advanced study showed that up-regulation of miR-143 expression promotes cancer cell invasion/migration and tumor metastasis by repression of FNDC3B expression [34]. The association between miR-215 and cancer were also reported. Senanayake et al. reported that miR-215 had a significantly lower expression in nephroblastomas regardless of the subtype compared with mature kidney measured by quantitative real-time-PCR [35]. White et al. performed experimental and bioinformatic analyses to explore the involvement of miR-215 in renal cell carcinoma progression and metastasis. In vitro study showed that miR-215 might contribute to kidney cancer metastasis through different biological processes [36]. In a previous study, miR-215 was reported to be up-regulated in HCC cancer cases [37]. Ishida et al. reported that using miRNA array analysis, miR-192/miR-215, miR-194, miR-320, and miR-491 were identified as being altered by HCV infection. Among them, miR-192/miR-215 and miR-491 were capable of enhancing replication of the HCV replicon as well as HCV itself [38]. To our best knowledge, this is the first study that reports the association between miR-143/miR-215 and HCC. More related studies are wanted to investigate to clinical application and detailed mechanisms.

Recent years, more and more studies raised diagnostic and prognostic application of miRNAs in different diseases, including cancer. Their application to body specimens from blood to tissues has been helpful for appreciating their use in the clinical context [39]. Our results showed that serum miR-143 and miR-215 were commonly up-regulated in chronic hepatitis and HCC patients, suggesting miR-143 and miR-215 may be new potential diagnostic biomarkers and targets of chronic hepatitis and HCC. However, as a case–control study without a long-time follow-up, the prognostic effect of the two miRNAs could not be detected in this study. A follow-up of this cohort would be reportedand more advanced analyses would be conducted.

## Conclusion

In conclusion, the expression of miR-143 and miR-215 in serum were significantly up-regulated in patients with chronic hepatitis and HCC. Due to its reasonable sensitivity and specificity for both diseases, miR-143 and miR-215 could be as potential circulating biomarkers. Our work will serve as a basis for further investigation, preferably large-scale validation in clinical trials, before serum miRNAs can be used as a routine screening tool for HCC.

## Competing interests

The authors declare that they have no competing interests.

## Authors’ contributions

ZQZ, HM, NW, LNL, SML and YL provided the conduction of the whole project, ZQZ, HM, SML and YL drafted the manuscript; ZQZ, HM, LNL, SML and YL contributed to revise the manuscript. All authors read and approved the final manuscript.

## References

[B1] Milanizadeh S Khanyaghma M Haghighi MM Mohebbi S Damavand B Almasi S Azimzadeh P Zali M Molecular analysis of imperative polymorphisms of MLH1 gene in sporadic colorectal cancer Cancer Biomark 2013 13 427 432 24595079 10.3233/CBM-140391 PMC12928317

[B2] Abdel-Wahab M El-Ghawalby N Mostafa M Sultan A El-Sadany M Fathy O Salah T Ezzat F Epidemiology of hepatocellular carcinoma in lower Egypt, Mansoura Gastroenterology Center Hepatogastroenterology 2007 54 157 162 17419252

[B3] Bao YX Cao Q Yang Y Mao R Xiao L Zhang H Zhao HR Wen H Expression and prognostic significance of Golgiglycoprotein73 (GP73) with Epithelial-mesenchymal transition (EMT) related molecules in Hepatocellular Carcinoma (HCC) Diagn Pathol 2013 8 197 24313979 10.1186/1746-1596-8-197 PMC3924912

[B4] Kojiro M Kawabata K Kawano Y Shirai F Takemoto N Nakashima T Hepatocellular carcinoma presenting as intrabile duct tumor growth: a clinicopathologic study of 24 cases Cancer 1982 49 2144 2147 6280834 10.1002/1097-0142(19820515)49:10<2144::aid-cncr2820491026>3.0.co;2-o

[B5] Volk ML Hernandez JC Su GL Lok AS Marrero JA Risk factors for hepatocellular carcinoma may impair the performance of biomarkers: a comparison of AFP, DCP, and AFP-L3 Cancer Biomark 2007 3 79 87 17522429 10.3233/cbm-2007-3202

[B6] Chen MH Jan YH Chang PM Chuang YJ Yeh YC Lei HJ Hsiao M Huang SF Huang CY Chau GY Expression of GOLM1 correlates with prognosis in human hepatocellular carcinoma Ann Surg Oncol 2013 20 Suppl 3 S616 S624 23838921 10.1245/s10434-013-3101-8

[B7] Dogan E Yalcin S Koca D Olmez A Clinicopathological characteristics of hepatocellular carcinoma in Turkey Asian Pac J Cancer Prev 2012 13 2985 2990 22938494 10.7314/apjcp.2012.13.6.2985

[B8] Huang J Deng Q Wang Q Li KY Dai JH Li N Zhu ZD Zhou B Liu XY Liu RF Fei QL Chen H Cai B Xiao HS Qin LX Han ZG Exome sequencing of hepatitis B virus-associated hepatocellular carcinoma Nat Genet 2012 44 1117 1121 22922871 10.1038/ng.2391

[B9] Zhou G Chiu D Qin D Niu L Cai J He L Tan D Xu K Expression of CD44v6 and integrin-beta1 for the prognosis evaluation of pancreatic cancer patients after cryosurgery Diagn Pathol 2013 8 146 24004467 10.1186/1746-1596-8-146 PMC3846138

[B10] Hansen TB Jensen TI Clausen BH Bramsen JB Finsen B Damgaard CK Kjems J Natural RNA circles function as efficient microRNA sponges Nature 2013 495 384 388 23446346 10.1038/nature11993

[B11] Su ZX Zhao J Rong ZH Geng WM Wu YG Qin CK Upregulation of microRNA-25 associates with prognosis in hepatocellular carcinoma Diagn Pathol 2014 9 47 24593846 10.1186/1746-1596-9-47 PMC4016611

[B12] Gargalionis AN Basdra EK Insights in microRNAs biology Curr Top Med Chem 2013 13 1493 1502 23745801 10.2174/15680266113139990098

[B13] Zhao J Lu Q Zhu J Fu J Chen YX Prognostic value of miR-96 in patients with acute myeloid leukemia Diagn Pathol 2014 9 76 24678958 10.1186/1746-1596-9-76 PMC3975266

[B14] Liu F Xiong Y Zhao Y Tao L Zhang Z Zhang H Liu Y Feng G Li B He L Ma J Qin S Yang Y Identification of aberrant microRNA expression pattern in pediatric gliomas by microarray Diagn Pathol 2013 8 158 24053158 10.1186/1746-1596-8-158 PMC3853583

[B15] Wang W Li F Zhang Y Tu Y Yang Q Gao X Reduced expression of miR-22 in gastric cancer is related to clinicopathologic characteristics or patient prognosis Diagn Pathol 2013 8 102 23786758 10.1186/1746-1596-8-102 PMC3733645

[B16] Li Y Chen L Chan TH Guan XY Hepatocellular carcinoma: transcriptome diversity regulated by RNA editing Int J Biochem Cell Biol 2013 45 1843 1848 23748106 10.1016/j.biocel.2013.05.033

[B17] Ronald JA Katzenberg R Nielsen CH Jae HJ Hofmann LV Gambhir SS MicroRNA-regulated non-viral vectors with improved tumor specificity in an orthotopic rat model of hepatocellular carcinoma Gene Ther 2013 20 1006 1013 23719066 10.1038/gt.2013.24 PMC3864878

[B18] Luo J Chen M Huang H Yuan T Zhang M Zhang K Deng S Circulating microRNA-122a as a diagnostic marker for hepatocellular carcinoma Onco Targets Ther 2013 6 577 583 23723713 10.2147/OTT.S44215 PMC3666878

[B19] Liu AM Yao TJ Wang W Wong KF Lee NP Fan ST Poon RT Gao C Luk JM Circulating miR-15b and miR-130b in serum as potential markers for detecting hepatocellular carcinoma: a retrospective cohort study BMJ Open 2012 2 e000825 10.1136/bmjopen-2012-000825 PMC3308260 22403344

[B20] Cui M Xiao Z Sun B Wang Y Zheng M Ye L Zhang X Involvement of cholesterol in hepatitis B virus X protein-induced abnormal lipid metabolism of hepatoma cells via up-regulating miR-205-targeted ACSL4 Biochem Biophys Res Commun 2014 445 651 655 24576478 10.1016/j.bbrc.2014.02.068

[B21] Gyugos M Lendvai G Kenessey I Schlachter K Halasz J Nagy P Garami M Jakab Z Schaff Z Kiss A MicroRNA expression might predict prognosis of epithelial hepatoblastoma Virchows Arch 2014 464 419 427 24570391 10.1007/s00428-014-1549-y

[B22] Li J Shi W Gao Y Yang B Jing X Shan S Wang Y Du Z Analysis of microRNA expression profiles in human hepatitis B virus-related hepatocellular carcinoma Clin Lab 2013 59 1009 1015 24273923 10.7754/clin.lab.2012.120901

[B23] Xiao F Zhang W Zhou L Xie H Xing C Ding S Chen K Zheng S microRNA-200a is an independent prognostic factor of hepatocellular carcinoma and induces cell cycle arrest by targeting CDK6 Oncol Rep 2013 30 2203 2210 24009066 10.3892/or.2013.2715

[B24] Yu L Ding GF He C Sun L Jiang Y Zhu L MicroRNA-424 is down-regulated in Hepatocellular Carcinoma and suppresses cell migration and invasion through c-Myb PLoS One 2014 9 e91661 24675898 10.1371/journal.pone.0091661 PMC3968007

[B25] Wang L Yao M Dong Z Zhang Y Yao D Circulating specific biomarkers in diagnosis of hepatocellular carcinoma and its metastasis monitoring Tumour Biol 2014 35 9 20 24006223 10.1007/s13277-013-1141-0 PMC3907675

[B26] Yang Y Gu X Zhou M Xiang J Chen Z Serum microRNAs: a new diagnostic method for colorectal cancer Biomed Rep 2013 1 495 498 24648974 10.3892/br.2013.109 PMC3917018

[B27] Sun L Hu J Xiong W Chen X Li H Jie S MicroRNA expression profiles of circulating microvesicles in hepatocellular carcinoma Acta Gastroenterol Belg 2013 76 386 392 24592541

[B28] Fu Y Wei X Tang C Li J Liu R Shen A Wu Z Circulating microRNA-101 as a potential biomarker for hepatitis B virus-related hepatocellular carcinoma Oncol Lett 2013 6 1811 1815 24260081 10.3892/ol.2013.1638 PMC3834113

[B29] Ng EK Li R Shin VY Siu JM Ma ES Kwong A MicroRNA-143 is downregulated in breast cancer and regulates DNA methyltransferases 3A in breast cancer cells Tumour Biol 2014 35 2591 2598 24218337 10.1007/s13277-013-1341-7

[B30] Naito Y Sakamoto N Oue N Yashiro M Sentani K Yanagihara K Hirakawa K Yasui W MicroRNA-143 regulates collagen type III expression in stromal fibroblasts of scirrhous type gastric cancer Cancer Sci 2014 105 228 235 24283360 10.1111/cas.12329 PMC4317817

[B31] Schee K Lorenz S Worren MM Gunther CC Holden M Hovig E Fodstad O Meza-Zepeda LA Flatmark K Deep sequencing the MicroRNA Transcriptome in Colorectal Cancer PLoS One 2013 8 e66165 23824282 10.1371/journal.pone.0066165 PMC3688869

[B32] Liu SG Qin XG Zhao BS Qi B Yao WJ Wang TY Li HC Wu XN Differential expression of miRNAs in esophageal cancer tissue Oncol Lett 2013 5 1639 1642 23761828 10.3892/ol.2013.1251 PMC3678876

[B33] Masamune A Nakano E Hamada S Takikawa T Yoshida N Shimosegawa T Alteration of the microRNA expression profile during the activation of pancreatic stellate cells Scand J Gastroenterol 2014 49 323 331 24404812 10.3109/00365521.2013.876447

[B34] Zhang X Liu S Hu T He Y Sun S Up-regulated microRNA-143 transcribed by nuclear factor kappa B enhances hepatocarcinoma metastasis by repressing fibronectin expression Hepatology 2009 50 490 499 19472311 10.1002/hep.23008

[B35] Senanayake U Das S Vesely P Alzoughbi W Frohlich LF Chowdhury P Leuschner I Hoefler G Guertl B miR-192, miR-194, miR-215, miR-200c and miR-141 are downregulated and their common target ACVR2B is strongly expressed in renal childhood neoplasms Carcinogenesis 2012 33 1014 1021 22431721 10.1093/carcin/bgs126

[B36] White NM Khella HW Grigull J Adzovic S Youssef YM Honey RJ Stewart R Pace KT Bjarnason GA Jewett MA Evans AJ Gabril M Yousef GM miRNA profiling in metastatic renal cell carcinoma reveals a tumour-suppressor effect for miR-215 Br J Cancer 2011 105 1741 1749 22033272 10.1038/bjc.2011.401 PMC3242591

[B37] Gui J Tian Y Wen X Zhang W Zhang P Gao J Run W Tian L Jia X Gao Y Serum microRNA characterization identifies miR-885-5p as a potential marker for detecting liver pathologies Clin Sci (Lond) 2011 120 183 193 20815808 10.1042/CS20100297 PMC2990200

[B38] Ishida H Tatsumi T Hosui A Nawa T Kodama T Shimizu S Hikita H Hiramatsu N Kanto T Hayashi N Takehara T Alterations in microRNA expression profile in HCV-infected hepatoma cells: involvement of miR-491 in regulation of HCV replication via the PI3 kinase/Akt pathway Biochem Biophys Res Commun 2011 412 92 97 21802413 10.1016/j.bbrc.2011.07.049

[B39] Chen Q Ge X Zhang Y Xia H Yuan D Tang Q Chen L Pang X Leng W Bi F Plasma miR-122 and miR-192 as potential novel biomarkers for the early detection of distant metastasis of gastric cancer Oncol Rep 2014 31 1863 1870 24481716 10.3892/or.2014.3004

